# Rapid and simple detection of *Glaesserella parasuis* in synovial fluid by recombinase polymerase amplification and lateral flow strip

**DOI:** 10.1186/s12917-019-2039-x

**Published:** 2019-08-14

**Authors:** Ting-ting Zhang, Meng-zhi Liu, Rong-huan Yin, Long-quan Yao, Bao-shan Liu, Ze-liang Chen

**Affiliations:** Key Laboratory of Livestock Infectious Diseases in Northeast China, Ministry of Education, College of Animal Science and Veterinary Medicine, Shenyang Agricultural University, No. 120 Dongling road, Shenyang, 110866 China

**Keywords:** *G. Parasuis*, Recombinase polymerase amplification, Point-of-care diagnosis

## Abstract

**Background:**

*Glaesserella parasuis* (*G. parasuis*) is an influential pathogen of the pig, which induces high morbidity and mortality in naive pig populations in the pig industry. Accurate and rapid detection of the agent is important for disease control. In this study, a simple recombinase polymerase amplification (RPA) with a Lateral flow (LF) strip (RPA-LF-GPS) was developed to detect *G. parasuis*.

**Results:**

The RPA-LF-GPS can specifically detect *G. parasuis* a limit of 100 CFU from other common related pathogens causing arthritis in the pig. The RPA-LF-GPS assay can use boiled synovial fluid samples as a template with the same sensitivity as other DNA extraction methods. In the detection of clinic positive synovial fluid sample, RPA-LF-GPS is equally sensitive (98.1%) compared with that of PCR (90.4%) (*P* > 0.05). The whole procedure of the RPA-LF-GPS assay could be finished in 1 hour without professional equipment.

**Conclusions:**

RPA-LF-GPS assay is a rapid and simple method for point-of-care diagnostic testing for *G. parasuis* infection.

## Background

*Glaesserella parasuis* disease, also known as Glaesser’s disease, is a common disease of pig caused by *G. parasuis* (GPS) previously known as *Haemophilus parasuis*, which is characterized by early colonization of the porcine upper respiratory tract [1] and difficult to be controlled by management procedures such as segregated early weaning. Glaesser’s disease occurs in pigs aged 4–12 weeks [2]. With the rapid development of the pig industry, it is becoming more and more widespread and causing huge economic losses to the pig industry.

*G. parasuis* causes serious systemic disease, such as fibrinous polyserositis, arthritis, and meningitis [3], the incidence rate of which is generally between 10 and 15%. Arthritis is a very common symptom of GPS infection. However, infections by other related pathogens, such as *Streptococcus suis* [4], *Erysipelothrix rhusiopathiae* [5, 6], *Mycoplasma hyorhinis* [7], *Actinobacillus pleuropneumoniae* [8] *and Actinobacillus suis* [9], also result in identical arthritis. Therefore, it is necessary to distinguish GPS infection from those caused by other pathogens allowing targeted treatment of the disease.

Early and rapid diagnosis is crucial for the prevention and control of *G. parasuis*. At present, preliminary clinical diagnosis based on the prevalence, clinical features, and pathological changes is not enough for confirmatory diagnosis. More precise diagnostic methods, including bacterial isolation and identification, molecular amplification, are required. Molecular methods have great application potential because of their rapidity, sensitivity, and specificity. Several molecular detection methods, including PCR [10, 11] and real-time PCR [12], have been developed for GPS diagnosis. However, these technologies require complicated instruments and skilled professionals, restricting their use in pig farms and veterinary clinics [13]. Simpler and convenient techniques were urgently needed for point-of-care diagnosis for GPS in field conditions.

Recombinase polymerase amplification (RPA) assay is an isothermal nucleic acid amplification technique established by Piepenburg et al. [14]. It has been explored for the molecular detection of diverse pathogens, e.g., bacteria [15], fungi [16], parasites [17, 18] and viruses [19, 20]. The technology has advantages of easy to operate, low equipment requirements and short inspection time, making it an ideal technique for point-of-care diagnosis. In this study, a novel RPA-based method was developed for rapid detection of *G. parasuis* for veterinary clinics.

## Results

### Development of RPA-LF-GPS assay

**Fig. 1:**
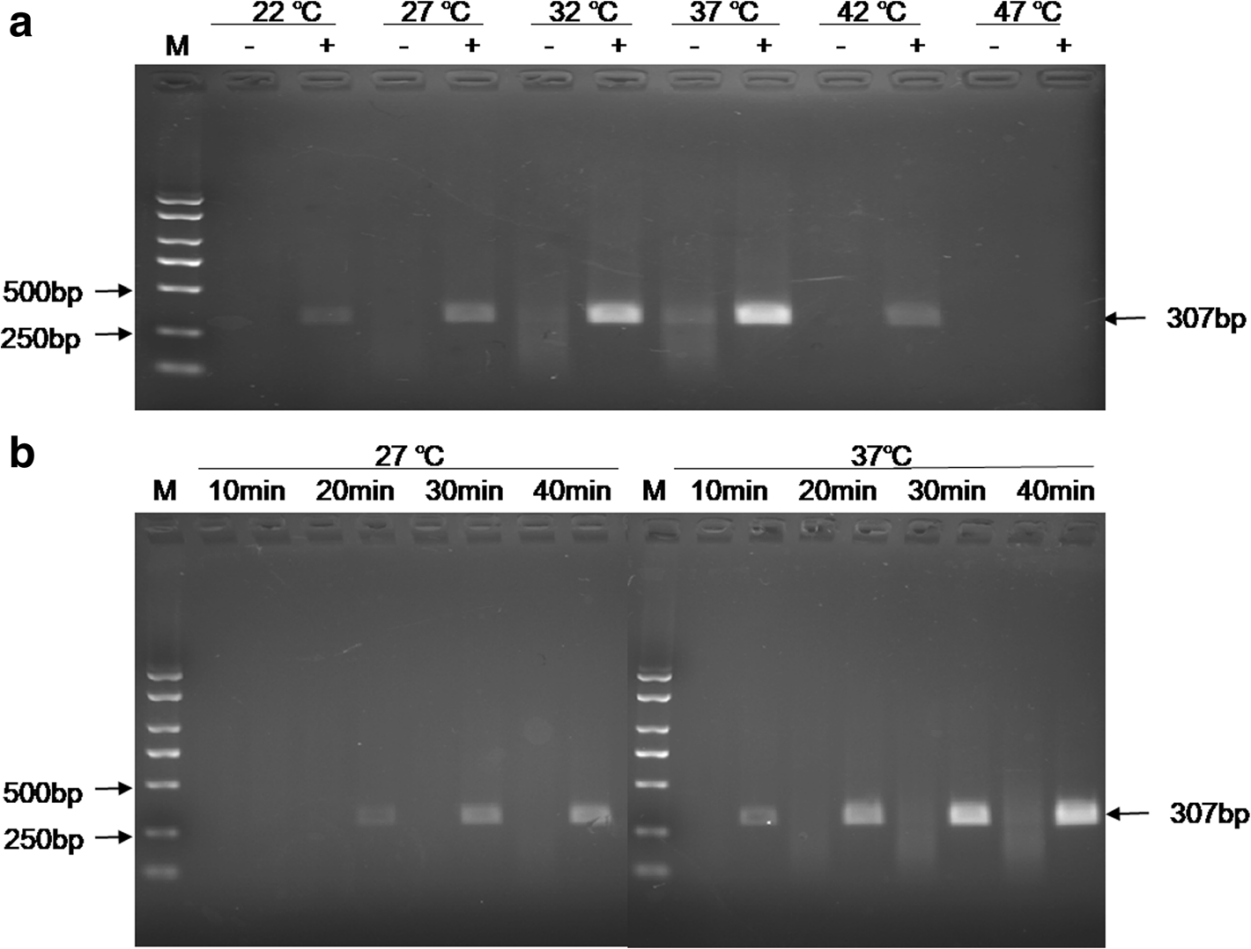
Optimization of RPA reaction. **a** RPA amplification under different temperature for 40 min. **b** RPA amplification with different incubation time. M. DL2000 plus DNA Marker

The RPA assay was performed under different temperature and time to optimize the reaction condition. After agarose gel electrophoresis and staining, RPA product with the expected size (307 bp) was clearly visible in the temperature range of 22–42 °C. Amplification in the temperature range of 32–42 °C generated more products, indicating this temperature range is optimal for the RPA assay (Fig. 1a). Semi-quantification by measuring the DNA band density revealed that the DNA yield at 37 °C was highest, so all RPA reactions were performed subsequently at 37 °C for the rest of this study. Then the RPA assay was performed at different length of time to optimize the reaction time. The amplification products could be detected on agarose gel at an incubation time as short as 10 min. However, when the incubation time was 20 min, clear bands were seen (Fig. 1b). Therefore, the final optimal condition of RPA assay was incubation at 37 °C for 20 min.

In fact, for evaluating the performation of RPA at room temperature, the assay was also performed at 27 °C. The time when bands were seen is at 20 min (Fig. 1b). which is twice as long than at 37 °C incubation. It showed that more amplification time is needed to obtain visible products at lower incubation temperature. This could be because the enzyme activity is lower under this unsuitable temperature.

### Sensitivity of RPA-LF-GPS

**Fig. 2:**
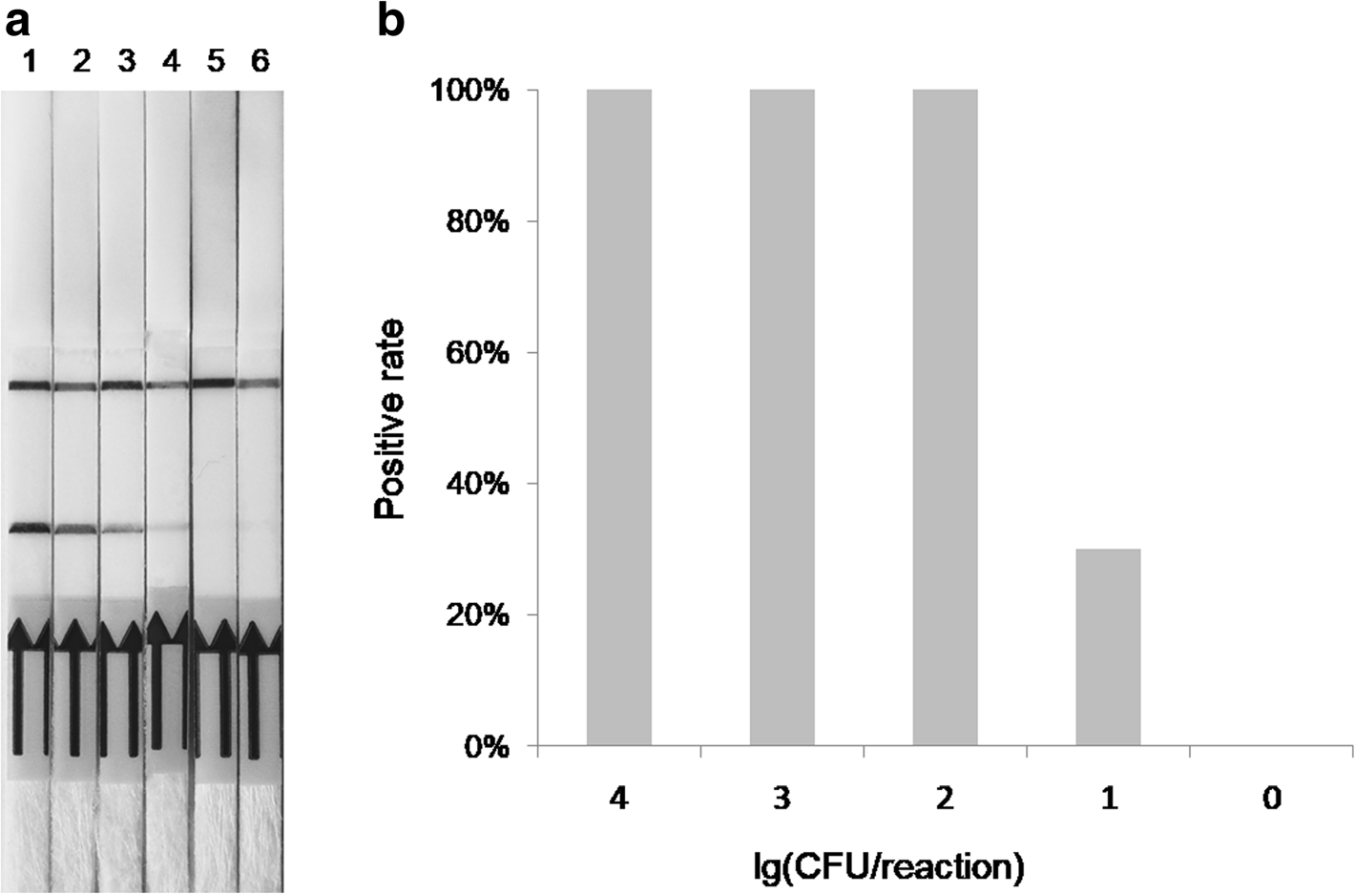
Sensitivity of RPA-LFD assay. **a** Different concentrations of GPS DNA were detected by RPA-LF-GPS. 1, 10^4^ copies; 2, 10^3^ copies; 3, 10^2^ copies; 4, 10 copies; 5, 1 copies; 6, negative control. **b** RPA-LF-GPS results in 10 repeat detection. The x-axis represents the log10 copies per reaction showing the percent of 10 reactions with the same DNA concentration that resulted in the same positive amplification result

The RPA assay was performed with serially diluted (10^4^–1 copies per reaction) GPS DNA. The result showed that positive detection could be obtained in 10 repeat detection when the concentration of template DNA is 100 or more copies per reaction (Fig. 2a). The detection with 10 copies of GPS DNA sometimes showed a light red test line, but the rate of detection was only 30% (Fig. 2b). Therefore, the sensitivity of the RPA-LF-GPS assay is 100 copies.

### Specificity of RPA-LF-GPS

The specificity of the RPA-LF-GPS assays was determined by GPS and related bacterial pathogens that also cause identical symptoms in pigs and are common in the piggery (Table 1). Detection of GPS generated positive results, while none of the other bacteria generated positive results, indicating that RPA-LF-GPS could specifically detect GPS and do not cross-react with related bacterial pathogens.

**Table 1:** Bacteria strains tested in this study and results detected by RPA-LF

Bacterial species	Strain ID	Source	Location isolated	RPA-LF	
				20 min	60 min
*Glasserella parasuis*	FS3	Pig (Fushun city)	synovial fluid	+	+
*Erysipelothrix rhusiopathiae*	HC8	Pig (Haicheng city)	synovial fluid	–	–
*Salmonella choleraesuis*	LY5	Pig (Liaoyang city)	enteric canal	–	–
*Escherichia coli*	SY13	Pig (Shenyang city)	Skin	–	–
*Staphylococcus aureus*	SY18	Pig (Shenyang city)	skin	–	–
*Mycoplasma hyopneumoniae*	TL4	Pig (Tieling city)	lung	–	–
*Pasteurella multocida*	DL6	Pig (Dalian city)	lung	–	+
*Brucella suis bv. 1 str. S2*	CVCC70502	Tecon Biology CO. Ltd		–	–
*Streptococcus suis type 2*	CVCC3306	CVCC		–	–
*Streptococcus suis type 1*	ATCC 43765	Beijing Zhongyuan Ltd.		–	–
*Actinobacillus pleuropneumoniae*	CVCC 261	CVCC		–	+
*Mycoplasma hyorhinis*	CVCC 361	CVCC		–	–
*Mycoplasma hyosynoviae*	ATCC 25591	Beijing Zhongyuan Ltd.		–	–

While in the test with 60 min incubation time, faint lines were visible in the dipsticks for *Pasteurella multocida and Actinobacillus pleuropneumoniae*. This indicated that false positive results appear in prolonged incubation time. The reason may be that the primers or probes have relatively high similarity to the genes of these bacteria which results in the wrong amplification after a long incubation time.

### Simplified procedure for RPA-LF-GPS

To make the RPA-LF-GPS appropriate for point-of-care diagnosis, a simplified DNA extraction procedure was tested. Simulated synovial fluid samples with known quantities of bacteria were extracted with commercial kits or boiling treatment and then detected with the RPA-LF-GPS assay. The detection limits of RPA-LF-GPS assay for extracted DNA and boiled supernatant were 100 CFU per reaction where the results of both assays were mostly positive in the 10 repetitive tests (Table 2) (*P* > 0.05). This indicated that boiling is a feasible sample treatment procedure to replace the extracted procedure in the RPA-LF-GPS assay.

**Table 2:** Simplified procedure of spiked samples

CFU/reaction	Extracted DNA	Boiling supernatant	coincidence rate
10^3^	10/10	10/10	100%
10^2^	8/10	6/10	80%
10	1/10	0/10	90%

### Evaluation with clinical samples

A total of 52 synovial fluid samples subjected to GPS infection were detected respectively with RPA-LF-GPS and PCR test [21]. Of these samples, 51 were positive by RPA-LF-GPS, and 47 positives by PCR, generating a positive rate of 98.1 and 90.4% respectively (Table 3). Four inconsistent samples positive by RPA-LF-GPS but negative by PCR were confirmed by DNA sequencing of amplification products (Table 4). The results showed that these samples (Synovial fluid 2, 11, 31, 36 and 45) were indeed real positives. Another positive sample was tested negative by PCR and RPA-LF-GPS (Table 4), which might have resulted from long term storage of the sample. However, the Chi-square test shows that there is no significant difference in detection rate between the two methods (*P* > 0.05).

**Table 3:** RPA-LF and PCR assay of 52 clinical positive samples

Assay	Negative	Positive	Detection rate
RPA	1	51	98.1%
PCR	5	47	90.4%

**Table 4:** Synovial fluid samples which gave the inconsistent result of detection by Culture, PCR, and RPA-LF

Sample source	Gathering area	Culture	PCR	RPA-LF
Synovial fluid 2	Fushun city, Liaoning province, China	+	–	–
Synovial fluid 11	Liaoyang city, Liaoning province, China	+	–	+
Synovial fluid 31	Panjin city, Liaoning province, China	+	–	+
Synovial fluid 36	Haicheng city, Liaoning province, China	+	–	+
Synovial fluid 45	Chaoyang city, Liaoning province, China	+	–	+

## Discussion

*G. parasuis* infection is of considerable economic importance in the pig industry due to high morbidity and mortality in naive pig populations [22]. It is also widespread in the pig population of the pig industry of China, especially small size farms and home farms. Thus, a robust and simple diagnostic assay, which is capable of the rapid, sensitive and specific detection of GPS in these situations, could play a significant role in the reduction of the disease-related morbidity and mortality.

Molecular diagnostic techniques have received great emphasis in recent years due to the rapidity, specificity, and sensitivity. However, the need for specialized instruments for the extensively applied conventional PCR based techniques makes it difficult to be used in field condition. Isothermal amplification technology has advantages over PCR in terms of convenience and equipment requirement. Several isothermal techniques, including Loop-Mediated Isothermal Amplification (LAMP), Helicase-dependent amplification (HDA) and recombinase polymerase amplification (RPA), have been developed and applied for pathogen detection [23]. In these methods, RPA has been substantially applied for the detection of various animal pathogens by virtue of its advantages, such as simple operation, rapidness, and convenience, visual detection by lateral flow strip [24–26].

In this study, we have developed an RPA-LF-GPS assay for the detection of GPS using primers and probes targeting the *infB* gene. The sensitivity and specificity of assays were determined. Finally, we applied the assays to the detection of GPS directly in the boiled positive synovial fluid from pigs.

The RPA-LF-GPS assay had a reaction temperature range of 22–42 °C (Fig. 1a). Although performed better at 32–37 °C. A previous study has shown that body heat could be used as an incubator [27]. The feature of RPA-LF-GPS reaction under room temperature represents a breakthrough in terms of the amplification procedure. For it completely avoids incubation under the specific condition. RPA amplification products were detectable after 10 min of incubation and optimal amplification time was 20 min. This incubation time is consistent with previous reports [28].

The RPA-LF-GPS assay was highly sensitive with a detection limit of 10^2^ copies of GPS DNA. The detection line was faint at 10 min incubation time and could much more clearly seen at 20 min. This implied that there is a correlation between sensitivity and amplification time. However, the incubation time could not be extended unlimitedly, for nonspecific amplification generated when the incubation time was excessively extended. In our specificity analysis, we found that with routine incubation time, GPS but not other related pathogen generated positive amplification. However, when we extend the amplification to 60 min, other bacteria species also generate positive results. This nonspecific amplification might result from relatively high sequence homology of *infB* with other sequences or high amplification efficiency of RPA assay.

In order to prepare template easily of RPA-LF-GPS assay on farm, we test the boiling treatment procedure of synovial fluid. When bacteria were spiked into the synovial fluid, RPA-LF-GPS could detect as few as 100 CFU in the supernatant of the boiled synovial fluid, having the same sensitivity as using kit extracted DNA as a template. The high sensitivity of the simplified sample treatment procedure makes it valuable for field use. Boiling treatment avoids the use of specific equipment, making it possible to applying the assay at farm or other field situations.

Finally, the RPA-LF-GPS assay was evaluated with the clinical samples. The detection rate of RPA-LF-GPS is 98.1% (51/52), and that of PCR is 90.4% (47/52). Statistic analysis showed that RPA-LF-GPS and PCR had equal sensitivity (*P* > 0.05), which is consistent with previous reports about RPA [29]. This result showed that the RPA-LF-GPS performed well with clinic samples, which make it a potential to replace PCR. In addition, without special instruments and the steps of nucleic acid extraction and electrophoresis, the whole RPA-LF-GPS assay can be completed in less than an hour, greatly improving the speed of detection and broadening application places.

## Conclusion

A rapid, sensitive and specific RPA-LF-GPS targeting the *infB* gene was developed for detection of GPS in clinical synovial fluid samples. Simplified sample treatment, rapid amplification, and results observation by the naked eye make it an ideal assay for point-of-care diagnosis of GPS infection in field situations.

## Methods

### Bacterial strains, growth conditions and DNA extraction

The bacteria species used in the study are listed in Table 1. Some were isolated by our laboratory from pig farms in Liaoning province of China (marked in “source” column) and identified by their biochemical features and 16S rDNA sequencing. Others were purchased from China Veterinary Culture Collection Center, Tecon biology Co. Ltd. or Beijing Zhongyuan Ltd. respectively. Synovial fluids of pigs with GPS were collected from the slaughterhouses in Shenyang city and identified by GPS isolation in our laboratory. Positive synovial fluid samples were stored at − 80 °C. G. parasuis was cultured as described previously [22]. For bacterial enumeration, 100 μL of serially diluted bacterial suspension were plated on TSA and incubated at 37oC for 24 h. Bacterial genomic DNA was extracted using the Takara MiniBEST Bacteria Genomic DNA Extraction Kit Ver.3.0 (Takara Biotech Co. Ltd., Dalian, China) according to the manufacturers’ instructions. Extracted DNA was quantified by Nanodrop2000 (ThermoFisher, US) and stored in our laboratory.

### Primer and probe design

GPS *infB* gene sequence was obtained from GenBank (http://https://www.ncbi.nlm.nih.gov/nucleotide/) and aligned using the multiple sequence alignment tool ClustalW. GPS specific primers and probes for PCR and RPA were designed and screened for homology using the Prime-Blast (https://www.ncbi.nlm.nih.gov/tools/primer-blast/index.cgi?LINK_LOC=BlastHome. nih.gov/tools/primer-blast) (Table 5). The infBRPARB primers were labeled with 5′ biotin residues to adapt for the RPA-LF assay and an internal RPA-LF probe (infBRPAFB) was designed under suggestions of the TwistDX guidelines (Table 5). All oligonucleotide primers and probes were synthesized by Sangon Biotech Co. Ltd. (Shanghai, China).

**Table 5:** primer and probe sequence design for the GPS RPA and RPA-LF assays

Assay	Name	Sequence	Amplicon size
RPA	infBRPAF	CATTGAATCAGCWGGYCCATCAATTCCTGTGGA	307 bp
	infBRPAR	CACTTTTACTTCTGCCGTTGAAAGCTCGTGTAAA	
RPA-LF	infBRPARB	(B)CACTTTTACTTCTGCCGTTGAAAGCTCGTGTAAA	
	infBRPAFB	(F)ACTTTCAGGCGTACCAGCCGCAGGTGATGAAGC(H)GACAGTGGTGCGTGAT(3)	

Features: B = Biotin label; F = 6-carboxyfluorescein (FAM) label; H = abasic tetrahydrofuran (THF) residue; 3 = C3 spacer. All primers were designed according to the instructions from TwistDx (http://www.twistdx.co.uk)

### RPA assay development and optimization

RPA reactions were performed in a water bath with a TwistAmp nfo kit (TwistDx, UK). A total volume of 50 μL reagents contained 1X rehydration buffer and 5 μM each RPA primer (infBRPAF+ infBRPAR). Template DNA was added to the dry enzyme pellet and thoroughly mixed. Then the reaction was initiated by addition of 280 mM of magnesium acetate. The reactions were stopped by adding 50 μL of a mixture of chloroform/isoamyl alcohol (1:1). For electrophoresis analysis, the reaction products were centrifuged at 12,000 *g* for 1 min and 5ul of supernatant was electrophoresed on a 2.0% (w/v) agarose gel. To obtain optimize reaction conditions, the RPA assay was performed at 22, 27, 32, 37, 42 and 47 °C in the water bath. The reaction time was set at 10, 20, 30 and 40 min to optimize the reaction time. The amplified products were analyzed on a 2.0% (W/V) agarose gel.

### RPA-LF assay development

For RPA-LF-GPS assay, the reaction contained 5 μM each RPA primer (infBRPAF+infBRPAB), 0.5 μM of the LF probe (infBRPAP) and 0.25 M betaine (Sigma-Aldrich, UK). GPS DNA mixed with 1 x rehydration buffer and ddH_2_O was added to dry enzyme pellet and thoroughly mixed. The reaction was initiated by addition of 280 mM of magnesium acetate. After incubation, 0.2 μL of amplification products were diluted in 100 μL running buffer (Milenia Biotec, Germany) and Milenia Hybridetect (MGHD) Dipstrip (Milenia Biotec, Germany) was placed vertically into the running buffer. After 5 min at room temperature, the result was observed by naked eyes.

### Sensitivity and specificity analysis

Based on the length of the genome GPS and concentration of extracted DNA, the initial number of GPS DNA copies were calculated on the website: http://scienceprimer.com/copy-number-calculator-for-realtime-pcr calculator - for-realtime- pcr. The detection limit of the RPA-LF assays was determined by detection of serial dilutions of GPS genomic DNA. GPS DNA was diluted with ddH_2_O to reach a concentration of 10^4^–1 copies/μL and then used as a template for RPA assay. The specificity of RPA-LF-GPS was evaluated by detection of the DNA of a panel of related bacterial pathogens (Table 1). In addition to the 20 min incubation, a test with 60 min incubation was performed to determine the specificity of the RPA during the long incubation time.

### Procedure optimization

To develop a simple sample treatment procedure that could be performed at the point-of-care, boiling treatment procedure was developed for synovial fluid samples, a kind of relative easily acquired clinical samples of GPS. The bacterial culture was 10-fold serially diluted in TSA, and 2 μL aliquots were added into 98 μL fresh pig synovial fluid without GPS. Then the DNA was extracted from the spiked samples with the commercial Takara MiniBEST Bacteria Genomic DNA Extraction Kit Ver. 3.0 (Takara Biotech Co. Ltd., Dalian, China) and treated directly by boiling for 10 min. The kit extracted DNA was eluted with 100 μL ddH_2_O. The supernatant of the boiled sample was used as a template of RPA-LF-GPS. The detection result of the kit extracted DNA and boiling supernatant as a template was compared.

### Evaluation with the clinical samples

The synovial fluid samples stored in our laboratory were treated with commercial kit and boiling as described above. 1 μL extracted DNA or boiling supernatant was subjected to amplification by RPA-LF-GPS and PCR respectively.

## Data Availability

All data generated or analyzed during this study are available from the Corresponding author on reasonable request.

## Abbreviations

CFU: Colony-forming unit
CVCC: China Veterinary Culture Collection Center
ddH2O: Double distilled water
dH2O: Distilled water
DNA: Deoxyribonucleic acid
GPS: G. parasuis
LF: Lateral Flow Strip
PBS: Phosphate buffered saline
PCR: Polymerase chain reaction
RPA: Recombinase Polymerase Amplification
μL: Microliter
